# Regression-Based Analysis of Vestibular Laboratory Tests for the Prediction of Unilateral Vestibular Schwannoma

**DOI:** 10.1109/JTEHM.2026.3671170

**Published:** 2026-03-05

**Authors:** Giorgia Rita Di Ruggiero, Sarah Hösli, Christopher J. Bockisch, Julia Dlugaiczyk, Dominik Straumann, Carolina Beppi

**Affiliations:** Neuroscience Center ZürichUniversity of Zürich and ETH Zürich 8091 Zürich Switzerland; Interdisciplinary Center for Vertigo and Neurological Visual DisordersDepartment of NeurologyUniversity Hospital of Zürich (USZ) and University of Zürich 8091 Zürich Switzerland; Clinical Neuroscience CenterUniversity Hospital of Zürich (USZ) and University of Zürich 8091 Zürich Switzerland; Department of Otorhinolaryngology, Head and Neck SurgeryUniversity Hospital of Zürich (USZ) and University of Zürich 8091 Zürich Switzerland; Department of OphthalmologyUniversity Hospital of Zürich (USZ) 8091 Zürich Switzerland

**Keywords:** Diagnostics, machine learning, monocular vHIT, neurology, prediction, regression, vestibular schwannoma

## Abstract

Objective: The diagnostic work-up for vestibular pathologies involves a battery of tests designed to quantify the functioning of the otolith organs and semicircular canals. Clinical data from video head-impulse tests, vestibular-evoked myogenic potentials, subjective visual verticality, and caloric tests are usually collected. Our study applied regression analyses to predict the affected side of a patient group with vestibular schwannoma, learning from laboratory vestibular tests, to assess their relative predictive capacity in predicting the tumor side. Technology or Method: The dataset was pre-processed to handle missing values, outliers, and differences in the measurement scales. The mean asymmetry values and their direction (either negative = left-side asymmetry or positive = right-side asymmetry) were calculated. The classifiers’ ability to accurately predict the tumor side was evaluated. Finally, both logistic and multiple regression analyses were conducted. Results: The regression models’ binary output (i.e., right or left side affected) was compared to the true labels of the affected side given by magnetic resonance imaging to estimate the model’s accuracy. Linear regression analysis showed that caloric, cVEMP and RLLL reached AUCs >0.9; multiple regression revealed an AUC of 0.96 for caloric and cVEMP combined. Conclusion: Our study demonstrated that combining caloric and vestibular-evoked myogenic potential tests provides the most accurate identification of the vestibular schwannoma-affected side, achieving the highest predictive capacity. Furthermore, our findings align with previous studies revealing that the monocular video head-impulse test introduces a gain bias for all three semicircular canals that must be adjusted to correctly estimate semicircular canal function. Clinical and Impact—This study addresses the clinical challenge of finding the affected side in unilateral vestibular schwannoma patients by using machine learning to vestibular tests linking computational methods with clinical practice

## I. Introduction

Unilateral vestibular hypofunctions (UVH) selectively affect the functionality of the otolith organs and/or semicircular canals. These are commonly assessed with a battery of laboratory tests that quantify the asymmetry between the vestibular organs on the head’s left and right sides. Such tests typically include video head impulse testing (vHIT), vestibular evoked myogenic potentials (VEMPs), caloric irrigation and subjective visual vertical (SVV).

• The vHIT measures the vestibular ocular reflex (VOR) of all six semicircular canals (SCCs) [1], [2]. The VOR causes compensatory eye movements opposite to that of the head turns (head velocity between 150°/sec and 300°/s during head impulses [3]), to maintain the gaze stable on a target fixed on the wall [4]. For each semicircular canal, a gain is computed as the ratio of the area under the eye velocity and head velocity curve. Parameters of abnormality are VOR gain being lower than 0.8 for the lateral canals and VOR gain being lower than 0.7 for the vertical canals [5].
• The caloric test also measures the VOR [6]. It uniquely assesses the horizontal canals, albeit at a lower frequency range ( $\sim \!\!~0.003$ Hz) than the vHIT (up to 5 Hz) [7]. The vestibular system is stimulated by slow water irrigation of the auditory canal of both ears with water at 7°C below or above the body temperature (i.e., 30°C and 44°C) for about 30-40 seconds [8]. Caloric irrigation elicits compensatory eye movements (i.e., horizontal nystagmus) beating towards the side of the stimulated ear (warm water) or in opposite direction, away from the inhibited ear (cold water). The canal paresis (CP) is computed with the Jongkees’ formula [9] and is defined as “pathological” when an asymmetry >25% between the two sides is present [10].
• The VEMPs are short-latency muscle reflexes evoked by stimulation of the vestibular organs via air-conducted sound or bone-conducted vibrations. The cervical VEMPs (cVEMPs) mainly reflect ipsilateral saccular function, whereas the ocular VEMPs (oVEMPs) are mainly a measure of contralateral utricular function [11], [12].
• The SVV assesses the perceived verticality of a subject in total darkness. A subject is shown a line rotated to certain degrees to the left or right of the earth-vertical line and must reset it to perfect verticality (0°). This test is relevant in that pathological tilts of the SVV in the roll plane are frequent clinical signs of unilateral vestibular lesions [13]. This can be affected by acute otolith dysfunction [14] or by an acute unilateral lesion of central graviceptive pathways [15]. While the oVEMP and SVV both test utricular responses, they apply different stimulatory frequencies and are hence thought to evaluate different functional aspects (VEMPs: transient otolith function, SVV: sustained otolith function) [16], [17].

Discordant results exist in the literature as to the relative reliability of such laboratory vestibular tests in detecting conditions including Ménière’s disease [18], [19], [20], vestibular migraine [5], vestibular neuritis [7], [21], [22] and vestibular schwannoma [23], [24]. In clinical settings, determining the relative sensitivity and specificity of these tests for detecting vestibular dysfunctions can be difficult, particularly when diagnostic results are inconsistent, and clinicians must choose which test outcomes are most pertinent. In this study, we seek to assess the relative ability of such tests in predicting the affected side in a group of unilateral vestibular schwannoma (VS) patients by applying machine learning algorithms (MLAs). An MLA learns associations between data features and labels from historical data and uses them as input to classify or predict labels of values from new datasets [25]. In a clinical context, machine learning algorithms can serve as a valuable resource for evaluating the precision of specific screening tools in detecting a given pathology. Vestibular schwannoma (VS) is a benign, mostly slow-growing tumor that is diagnosed and monitored primarily with MRI. It arises from Schwann cells at the junction where the vestibulocochlear nerve’s (VCN, cranial nerve VIII) myelin sheaths transit from central to peripheral types [26], [27]. Abnormal Schwann cell growth compresses the proximal axon, forming a schwannoma. VS commonly compresses the cochlear nerve in the internal auditory canal (IAC), causing early hearing loss and tinnitus. As it enlarges, it may extend into the cerebellopontine angle (CPA), leading to balance disturbances, facial nerve and cerebellar symptoms [28].

Most cases are unilateral; bilateral cases may indicate neurofibromatosis type 2 [29]. VS impacts cochleovestibular function, with symptoms including hearing loss, tinnitus, imbalance, vertigo, and dizziness [30]. Larger tumors (>20 mm) can cause headaches, facial paresis, and cerebellar signs [31], [32]. They may also disrupt inner ear fluid regulation, leading to secondary endolymphatic hydrops [33]. Despite unilateral vestibular hypofunction, many patients do not experience subjective vestibular symptoms due to central vestibular compensation, especially in slow- growing tumors. Vestibular testing helps assess vestibular deficits and compensation, guiding management decisions (e.g., “wait and scan”, radiosurgery, microsurgery [34]).

Our investigation contributes to a body of research that has pioneered the use of machine learning algorithms to assess the relative informativeness of various vestibular tests in diagnosing distinct pathologies. An illustrative study by Priesol et al. [35] examines the diagnostic accuracy of vestibular test batteries in patients experiencing dizziness and imbalance. The study specifically evaluates two prevalent tests for lateral canal function: caloric and rotational testing. The authors conclude that rotational testing should supplant caloric testing for diagnosing unilateral peripheral vestibular damage, as it is nearly three times more effective. Another study by Anh et al. [36] assessed five supervised machine learning algorithms to effectively classify peripheral (PV) and non-peripheral (non-PV) vestibular diseases, using data collected from a sample of 1,009 patients. They observed that diagnostic accuracy improved to 83% for PV and 85% for non-PV when the predictions from all five machine learning models were consistent. The analysis of feature importance indicated that parameters derived from the caloric test, such as the canal paresis percentage, were the most significant factors in differentiating between the two disease groups.

In our study, we will consider a group of unilateral VS patients whose side of the lesion is known and provided by magnetic resonance imaging (MRI) evidence. We seek to estimate the predictive accuracy of logistic and multiple regression models learning from our standard laboratory vestibular tests. We hypothesized that the model would show accuracy and reveal which vestibular assessments are most reliable in identifying the tumor side.

## II. Methods

The study protocols (BASEC 2020-01569 and BASEC 2020-02853) were approved by the Ethics Committee of the Canton of Zurich. The experiments were conducted in accordance with the Declaration of Helsinki, the principles of Good Clinical Practice, the Human Research Act, and the Human Research Ordinance.

### A. Participants

The investigation was performed using data from two groups of subjects. The first group included 68 MRI-confirmed VS patients (32 females and 36 males, mean ± standard deviation (SD) age: $55~\pm ~14.96$ years) with variable tumor locations and sizes. The second group consisted of 33 subjects (26 females and 7 males, mean ± SD age: $42~\pm ~12.36$ years) affected by vestibular migraine [37] (VM), with normal vestibular function between the attacks, who acted as a control group selectively for the vHIT gain bias correction, as we will further detail in the Results section (“data pre-processing” subsection). All patient data were retrospectively extracted from the hospital information system. Inclusion and exclusion criteria are defined below:

Inclusion criteria–VS patient group: (1) MR-confirmed diagnosis; (2) Complete pre-operative vestibular testing (i.e. before undergoing any interventions). Comparison group (i.e., VM patients): (1) Fulfillment of diagnostic criteria [37] of VM by the Bárány Society; (2) Normal vestibular function between attacks, for all laboratory tests, using the pathological thresholds specified in section “Asymmetry definition”.

Exclusion criteria–VS patient group: (1) The VS patient is currently affected or was affected (i.e., clinical history) by an additional neurological, otological or vestibular condition. Comparison group (i.e., VM patients): (1) The subject with VM is currently affected or was affected (i.e., clinical history) by an additional neurological, otological or vestibular condition.

### B. Asymmetry Definition

For each laboratory vestibular test, a measure of right-left asymmetry was computed. The asymmetry measure for the semicircular canals is computed using the vHIT test, based on the gain of the six SCCs. In summary, gains are computed as the ratio between the eye velocity (°/s) and the head velocity(°/s) [38] (see Janky et al. [39] for different methods of gain calculation). The asymmetry for the lateral canals is defined as follows, where LL and RL indicate the left lateral and right lateral canals, respectively:

\begin{equation*} RLLL=\frac {LL~gain-RL~gain}{RL~gain+LL~gain}\cdot 100 \tag {1}\end{equation*}

While both lateral canals are situated in one plane, the situation is a bit more complicated for the vertical (anterior and posterior) canals, where the anterior canal of one side lies in the same plane as the posterior canal of the contralateral side, defining the RALP (right anterior–left posterior) and LARP (left anterior–right posterior) planes. Thus, the left-right asymmetry values for the anterior and posterior canals are defined as follows:

\begin{align*} RALP& =\frac {LP~gain-RA~gain}{RA~gain+LP~gain}\cdot 100 \tag {2}\\ LARP& =\frac {LA~gain-RP~gain}{LA~gain+RP~gain}\cdot 100 \tag {3}\end{align*}

Pathological thresholds for the vHIT measures are the following: for the lateral canals: gain <0.8; for each posterior and anterior canal: gain <0.7. Analyzing differences in vHIT gains can be challenging for three main reasons:

(1) Testing with monocular goggles introduces a bias between left and right lateral canals’ gain, at the expense of the non-measured side. This bias was first described by Weber at al. [40] and confirmed by Strupp et al. [41], who showed that if the VOR is recorded from the right eye, the left lateral canal’s gains would be lower than the right lateral canals’ gains. The authors discussed that such findings relate to differences in the trans-synaptic pathway of the signal when testing the right and left canals: the adduction pathway (left) includes one interneuron more than the abduction one [40], [41] (right), resulting in a delay. However, such left-right discrepancy seems to occur also for the anterior (i.e., LA-RA comparison) and posterior canals (i.e., LP-RP comparison), as we will detail in the Results section.
(2) vHIT gains are not adjusted for differences in mean head velocity/acceleration, introducing a component of variability between subjects that should not be overlooked. In fact, head acceleration strongly affects the gains, with higher head velocity resulting in lower gains in cases of vestibular hypofunction [2].
(3) Although the LARP and RALP metrics are analogous to the lateral canals in that an excitation on one side produces an inhibition on the contralateral side, the RLLL measure selectively assesses the lateral canals functionality, whereas the RALP and LARP measures combine two different structures (ipsilateral anterior canal and contralateral posterior canal), which are functionally distinguished. Hence, LARP and RALP could be considered as being more “mixed” metrics than the RLLL. While it is possible to obtain a selective quantification of the lateral canals’ functionality by mean of caloric testing, there is currently no technique providing an isolated measure for either both anterior or posterior canals (i.e., LARA and LPRP). In an attempt to increase the reliability of RALP and LARP to other asymmetry metrics (i.e., VEMPs, SVV, RLLL etc.), we will introduce in our analysis a “composite” RALP-LARP measure, which we will refer to as cmpAP, where the left anterior and posterior canals are compared to the right anterior and posterior canals. The cmpAP metric is obtained starting from two different “composite” measures of RALP and LARP:
\begin{align*} cmp_{A}& =\frac {LALP-RARP}{LALP+RARP}\cdot 100 \tag {4}\\ cmp_{B}& =\frac {RALP+LARP}{2}\cdot 100 \tag {5}\end{align*}

The formulas of RALP and LARP substituted in (5) are as follows:

\begin{align*} & =\frac {1}{2}\left [{{ \frac {LP\!-\!RA}{LP\!+\!RA}\!+\!\frac {LA\!-\!RP}{LA\!+\!RP} }}\right ]\cdot 100 \\ & =\frac {1}{2} \\ & \left [{{ \frac {LALP\!+\!RPLP\!-\!RALA\!-\!RARP\!+\!LALP\!-\!RPLP\!+\!RALA\!-\!RARP}{LALP\!+\!RPLP\!+\!RALA\!+\!RARP} }}\right ] \\ & \quad \cdot 100 \\ & =\frac {1}{2}\left [{{ \frac {2LALP\!-\!2RARP}{LALP\!+\!RPLP\!+\!RALA\!+\!RARP} }}\right ]\cdot 100 \\ & =\frac {LALP\!-\!RARP}{LALP\!+\!RPLP\!+\!RALA\!+\!RARP}\cdot 100\end{align*}

From this simplification, we can notice that cmpA is “contained” in cmpB, which has two additional variables (summed) in its denominator: RPLP and RALA. Our pilot analysis showed that these two measures (cmpA and cmpB) had different predictive abilities, with cmpB having a better classificatory ability than cmpA, suggesting that the better performance could be attributable to the additional variables in cmpB’s denominator. To finalize our “composite measure”, we extracted these additional variables and applied the Jongkees formula [9], [41] as follows:

\begin{equation*} cmpAP=\frac {RPLP-RALA}{RPLP+RALA}\cdot 100 \tag {6}\end{equation*}

The asymmetry value of the oVEMPs and cVEMPs is based on mean peak-to-peak amplitudes (oVEMP: n10p15; cVEMP: p13n23). The amplitude is computed by averaging two blocks of 150 or 200 trials and corrected for the muscular pre-tension in case of the cVEMPs. The asymmetry value is hence calculated using the following formula [39]:

\begin{equation*} AR=\frac {\left ({{ Right~Amplitude-Left~Amplitude }}\right)}{\left ({{ Right~Amplitude+Left~Amplitude }}\right)}\cdot 100 \tag {7}\end{equation*}

The caloric test provides a measure of asymmetry that considers both the mean frequency and the mean slow-phase velocity (SPV) of the thermally induced nystagmus in the cold (30°C) and warm (44°C) water irrigation conditions, for both the left and right ears (total = 4 conditions). The maximal slow phase velocity (SPV) of the nystagmus is determined. The left-right asymmetry value is computed by using the Canal Paresis (CP) formula (see below), which combines SPV values in the 4 conditions, applying the Jongkees formula [9], [42]:

\begin{align*} & CP\!=\!\frac {\left [{{ \left ({{ righ~tcold+right~warm }}\right)-\left ({{ left~cold+left~warm }}\right) }}\right ]}{\left ({{ right~cold+right~warm+left~cold+left~warm }}\right)} \\ & \qquad \cdot 100 \tag {8}\end{align*}

Here, “left warm” and “right warm” correspond to the mean SPV for left and right ears, respectively, in the warm water condition, while “left cold” and “right cold” are the mean SPV for the left and right ears, respectively, in the cold condition. CP can be calculated automatically with a commercial VNG system software (e.g., OtoAccess Micromedial VisualEyes ${^{\text {TM}}}~525$ by Interacoustics). A CP>25% asymmetry is considered an indicator of unilateral hypofunction of the lateral canal [12].

Finally, in the Subjective Visual Verticality (SVV) test, the patient must reset a randomly tilted line [14], [43] back to the earth-vertical axis, while in complete darkness. This test provides an estimation of the subject’s average shift (in degrees) in verticality perception. The mean asymmetry value is calculated by averaging the subject’s performance (i.e., shift from 0°) in six trials. Normal SVV degree-bias values range between −2.2° and 2.2°. This means, a negative perceived verticality shift $< \!\!- 2.2^{\circ }$ indicates a pathological left asymmetry, whereas a positive perceived verticality shift >2.2° indicates a pathological right asymmetry.

### C. Material and Technical Devices

Vestibular assessment was conducted using a standardized test battery. The video Head Impulse Test (vHIT) was performed with the ICS Impulse system (Natus Medical Inc., USA). Vestibular evoked myogenic potentials (VEMPs) were acquired with the Eclipse platform (Interacoustics A/S, Denmark). Caloric testing was carried out with the VisualEyes VNG system (Interacoustics A/S, Denmark). The subjective visual vertical (SVV) was evaluated using a custom device.

### D. Analytical Pipeline

**FIGURE 1.**
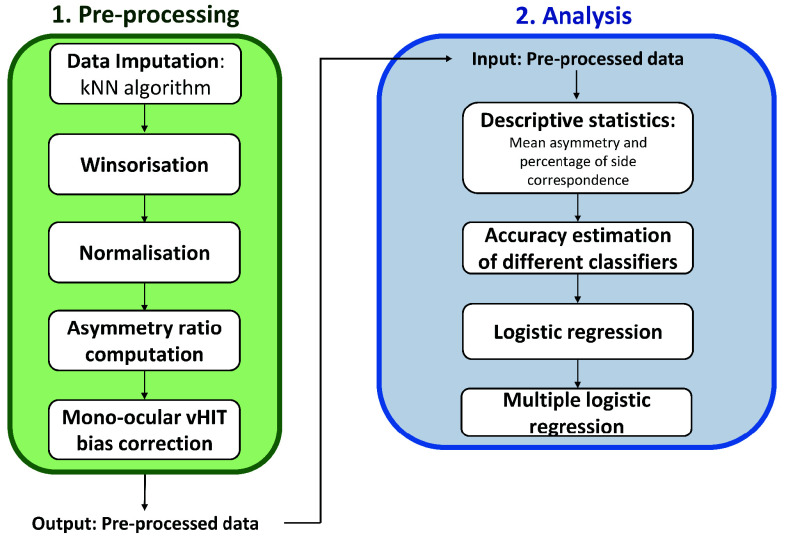
Flow diagram of the data analysis pipeline. Green box: description of pre-processing steps. Blue box: formal analysis steps with pre-processed data as input.

The data first underwent a pre-processing step to deal with missing values and outliers and to adjust for variability in measurement scales, as explained in the “Data pre-processing” section. Next, gains of all SCCs were corrected for the monocular vHIT bias [40], [41] as we will detail in section “Descriptive statistics”. Next, mean asymmetry values and the direction or sign of asymmetry (− or +) for the VS patients were computed, as we will explain in section “Descriptive statistics”. Different classifiers were then evaluated for the predictive accuracy in identifying the affected side of VS, as detailed in section “Regression-based predictions of the affected side”. Finally, logistic and multiple regression analyses were conducted for further analysis. All statistical methods, analyses and computations were performed using MATLAB software version R2022b (MathWorks, Natick, MA, USA). A schematic overview of our analytical pipeline is provided in Fig. 1.

### E. Data Pre-Processing

The clinical data for each test was pre-processed in three steps (Fig. 1): (1) data imputation for dealing with missing values, (2) winsorization for managing outliers and (3) normalization using the range of uppermost and lowermost values, applied per person. These pre-processing steps were performed within cross-validation folds.

The k-Nearest Neighbor (k-NN) algorithm was used to predict missing values [44]. This method is based on a distance measure (e.g., Minkowski, Euclidean) and a number of contributing Neighbors (k) for each prediction. Once the k-Neighbors are found, the prediction can be computed by combining all the k-Neighbours. In this analysis, we used the Euclidean distance measure and k= 1. The prediction is the value assumed by the Neighbour found. The hyperparameter k was fixed a priori, and no data-driven tuning was performed. 1-NN provided the best trade-off between discrimination and interpretability, minimizing bias and preserving local structure in a small-sample clinical dataset. Choosing higher k values, such as two or three, could lead to averaging and regression-to-the-mean effects, which might diminish the magnitude of asymmetry. Considering the limited sample size and the aim to retain physiological variability rather than smooth it out, using 1-NN was deemed the most suitable approach.

Eventual outliers (values beyond the mean +/- 2 SD) were winsorized [45], namely replaced with the minimum or maximum value (in the same direction) assumed by the metric within the acceptable range.

Normalization was performed using the range of uppermost and lowermost asymmetry values, for all vestibular tests:

\begin{equation*} D_{norm}\left ({{i,j }}\right)=\frac {D\left ({{i,j }}\right)}{max\left ({{ D\left ({{:,j }}\right) }}\right)-\left ({{ D\left ({{:,j }}\right) }}\right)} \tag {9}\end{equation*}

Here, $D\left ({{i,j }}\right)$ is the value of asymmetry metric $j$ for patient $i$.

### F. Descriptive Statistics

To obtain a realistic and reliable estimate of the asymmetry between left and right vHIT gains in VS patients, we developed an approach to retrospectively eliminate the gain bias introduced by the monocular vHIT goggles [40], [41]. The ideal approach to go about this would be to quantify the “offset”, namely the mean left-right asymmetry in a healthy control group, which is expected to have an approximately null left-right gain difference. As such, any observed gain asymmetries in this group would be attributable to the bias introduced by the monocular vHIT goggles. In absence of readily available vHIT data of an age-matched healthy control group, in our analysis, we calculated this offset in a comparison group of subjects with VM, whose vHIT gains were control-like based on clinical observation. The comparison group’s gain difference between sides (i.e., left canal’s gain minus right canal’s gain) was hence considered as an approximation of the “normative” gain bias (i.e., and hence considered as a reference value or offset). This offset was subtracted from the VOR gains of schwannoma patients to eliminate the bias from the VS patient group’s data, hence obtaining an “unbiased” asymmetry value estimation for all three SCCs. One-sample t-tests were finally used to determine the presence of any statistically significant differences between the right and left canal gains for all the SCCs (lateral, anterior, posterior), for the comparison group (anterior canals p= 0.35, lateral canals p<0.0001, posterior p= 0.036), and for the VS patient group, before and after gain correction.

The average asymmetry values (and their directions) for each laboratory vestibular test were computed. A positive asymmetry direction (i.e., left minus right equaling a positive value) indicates a better left side function. Vice versa, a negative asymmetry direction (i.e., left minus right equaling to a negative value) indicates a better right-side function. The affected side was labelled through a binary scoring. For example, if the laboratory test’s result of a patient (p) outputs a pathologically negative asymmetry value, p would be labelled as −1, meaning the left side is affected. Vice versa, if the test outputs a pathologically positive asymmetry value, p would be labelled as + 1, meaning the right side is affected. Such binary scores will be compared to the MRI-given true (+ 1 or −1) labels to compute the percentage of side correspondence. We intuitively hypothesized that if a given laboratory test has a high group-average asymmetry value and a high percentage of side correspondence, this test would likely make a good regressor (in identifying the affected side). It is possible that patients with VS show “opposite” asymmetry directions in certain laboratory tests (e.g., an SVV test with a positive asymmetry value in a patient with left-sided VS). There are three possible reasons (not mutually exclusive) for which this might be the case: (A) The patient has a pre-existing deficit to the side opposite to VSM, either congenital or slowly developed over time in a way that made it unnoticeable to the patient. (B) In rare cases, atypical findings were noted, such as increased vestibular-evoked myogenic potentials on the tumor side of the patients with intravestibular schwannomas [46], and an eye velocity response on the video head impulse test was also abnormally high in those patients with endolymphatic hydrops [47]. (C) There was a human error (in the conduction or interpretation) of the laboratory test results, which retrospectively we cannot derive. Such cases–which are asymmetries unexplained by VS–cannot be excluded in our sample. To understand to what extent mean group asymmetries (in terms of size) of our sample might be affected by such cases, we selected as a subgroup–which we will refer to as the “subset”–of subjects for whom the asymmetry direction corresponded to the MRI-given true label. Such subset was selected for each laboratory vestibular test, calculating its mean asymmetry value, and compared to the mean group asymmetry of the whole dataset, for all laboratory tests. Pearson’s test was conducted to assess the strength of correlation between the mean asymmetry value and the percentage of side correspondence, for each laboratory vestibular test, in the whole VS patient group. Our intent was to evaluate the algorithm’s ability to identify the VS side, despite of the presence of such cases. This means that the algorithm would be able to give more weight to VS-related asymmetries compared to VS-unrelated asymmetries. This subset was only used for descriptive purposes and was not included in ML analysis. In the following sections, we detail our algorithm-based predictive approach.

### G. Regression-Based Predictions of the Affected Side

The data was partitioned and cross-validated using the leave-one-out cross-validation (LOOCV) method. Then, Naïve Bayes, Support Vector Machine (SVM) and unregularized logistic regression, and SVM classifiers were compared for their average predictive accuracy. Since logistic regression showed a slight accuracy advantage over the other classifiers, only this method was used for the predictive analyses. The input values were the asymmetry values (standardized to zero mean and unit variance within each training fold) of each laboratory vestibular test, selected a priori based on physiological relevance, and the output values were the affected sides (right = 1 and left $= -1$) of each patient. The model output labels were then compared to the MRI-given true labels for an estimation of the model’s accuracy. Next, multiple regression was conducted to evaluate the predictive ability of the asymmetry metrics in a stepwise fashion, where metrics were ordered based on their weights, computed through an SVM. For SVM, hyperparameters were fixed a priori, and no data-driven tuning was performed. We assessed model performance using cross-validated, out-of-sample AUC, which inherently addresses model complexity by reducing overfitting. Finally, we considered which combinations of metrics achieve the highest predictive ability of VS side (Fig. 6). To do so, we examined the three vHIT measures (RLLL, RALP, LARP) altogether, as a single proxy of the semicircular canals’ functionality, and the combined oVEMP and cVEMP measures, as a proxy of the otolith’s functionality.

**FIGURE 2.**
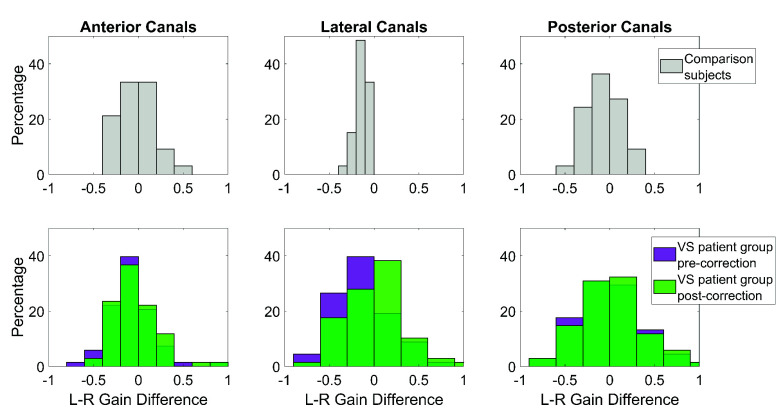
First row: distribution of “left minus right” gain differences for the anterior (left panel), lateral (middle panel) and posterior (right panel) SCCs of the comparison group. Second row: “left minus right” gain differences of VS patients before (purple) and after (green) gain bias correction. The y-axis corresponds to the percentage of patients within specific gain-difference ranges.

**FIGURE 3.**
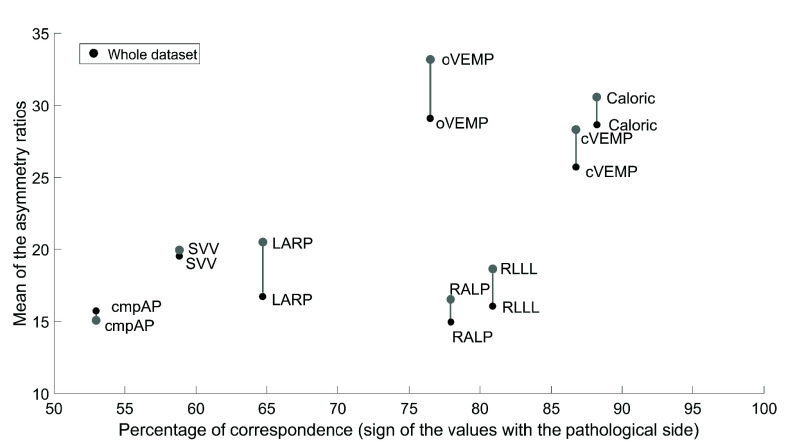
Mean asymmetry ratios (y-axis) and percentage of side matching (x-axis). For each metric, the mean asymmetry value in the entire dataset (N= 68) is indicated by a full black dot. The grey projections show the shift in mean asymmetry when considering the subgroup of VS patients (i.e., the “subset”) whose asymmetry direction or sign matches the MRI-given labels of the affected side.

**FIGURE 4.**
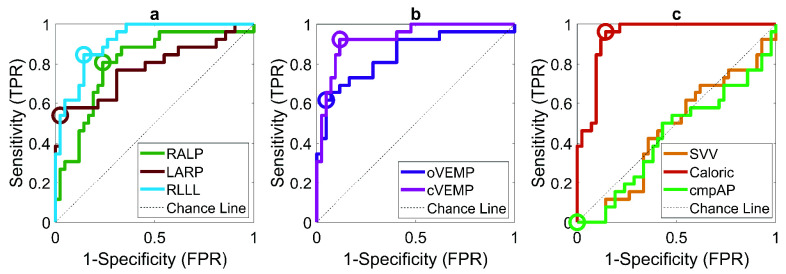
ROC curves obtained through logistic regression for each laboratory test metric: a) RALP, LARP and RLLL, b) oVEMP and cVEMP, c) SVV, Caloric and cmpAP. The open circle marks each ROC curve’s optimal operating point (OOP). AUC values (95%CI) for each ROC curve are: RALP 0.80(0.68-0.91), LARP 0.78(0.64-0.89), RLLL 0.92(0.84-0.97), oVEMP 0.84(0.74-0.94), cVEMP 0.93(0.86-0.98), SVV 0.46(0.33-0.60), Caloric 0.94(0.88-0.99), cmpAP 0.44(0.30-0.59). The OOP values for each ROC curve are: RALP 0.81, LARP 0.54, RLLL 0.85, oVEMP 0.61, cVEMP 0.92, SVV 0, Caloric 0.96, cmpAP 0.

**FIGURE 5.**
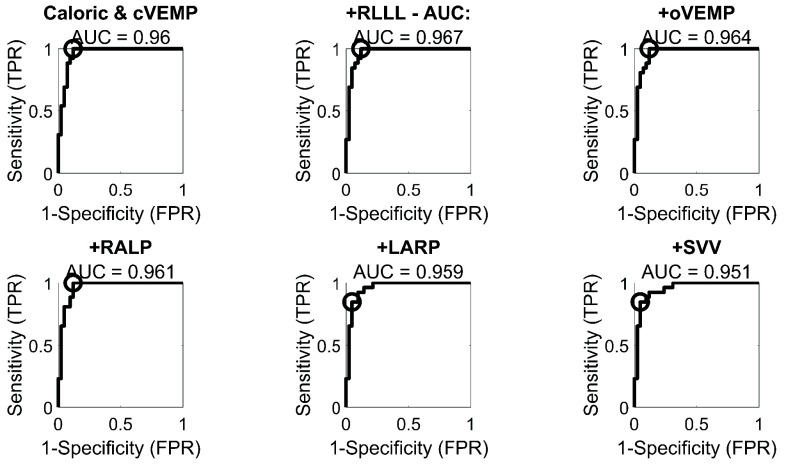
ROC curves obtained through multiple logistic regression. The 95% confidence intervals (CI) for each ROC curve are: Caloric and cVEMP: 0.91-0.99, +RLLL: 0.92-0.99, +oVEMP: 0.91-0.99, +LARP: 0.90-0.99, +RALP: 0.90-0.99, +SVV: 0.89-0.99. The circle indicates the optimal operating point (OOP) for each ROC curve. OOP values for each ROC curve are: Caloric and cVEMP 1 (sensitivity = 1, specificity = 0.88), + RLLL 1, + oVEMP 1, + RALP 1,+ LARP 0.84, + SVV 0.84.

**FIGURE 6.**
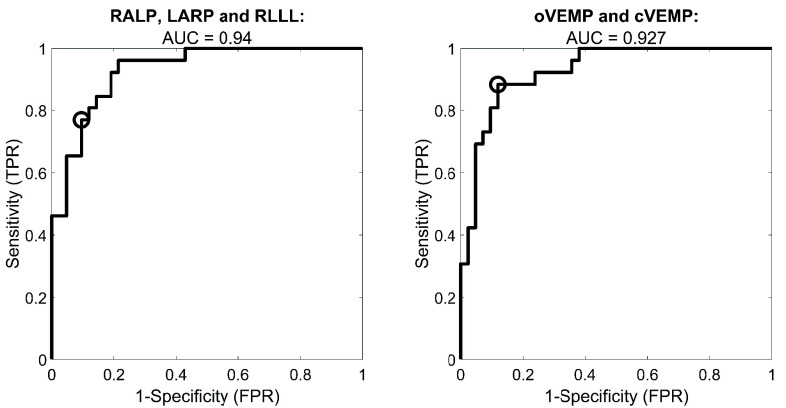
ROC curves obtained through multiple logistic regression. Left panel: vHIT metrics (RALP, LARP and RLLL). Right panel: VEMPs. The circle indicates the optimal operating point (OOP) for each ROC curve. The OOP values for each ROC curve are: RLLL, RALP &LARP 0.77, oVEMP & cVEMP: 0.88.

## III. Results

### A. Data Pre-Processing

Missing values per test were identified and replaced with k-NN algorithm. In percentage the missing values were: vHIT: RA = 0%; LA = 0%; RL = 0%; LL = 0%; RP = 0%; LP = 0%; oVEMP = 4.4%; cVEMP = 0%; SVV = 5.8%; Caloric = 7.3%. Outliers were identified for two out of the five laboratory tests and corrected through winsorization, as explained in the methods section. No test had more than five outliers. In percentage the outliers were: vHIT: RA = 5.8%; LA = 4.4%; RL = 5.8%; LL = 7.3%; RP = 4.4%; LP = 5.8%; SVV: 4.4%.

### B. Descriptive Statistics

Gain bias correction. The gain of the right SCCs was slightly higher than that of the left SCCs in both VS and comparison groups (Fig. 2). The average gain distribution for the lateral, anterior and posterior SCCs is summarized below for the comparison group (see Supplementary Table 1) and for the VS patient group before and after gain bias correction (see Supplementary Table 2). Supplementary Tables 1 and 2 also indicate significant differences in gain between the right and left sides, for each SCC.

Mean asymmetry and percentage side. After the pre-processing step and gain bias correction, the mean asymmetry values and the percentage of side correspondence (i.e., the percentage of VS patients whose asymmetry direction or sign corresponded to the MRI-given true label) were computed and are displayed in Fig. 3 for all the laboratory vestibular tests. The figure details the mean asymmetry value in the whole dataset of subjects, and the mean asymmetry value for the “subset” (i.e., the subgroup of VS patients whose asymmetry direction in a given laboratory test corresponded to the MRI-given true label). The metrics showing the highest combination of mean asymmetry and side correspondence were the caloric, cVEMP and oVEMP tests, whereas the lowest were cmpAP, SVV and LARP. The metrics showing the highest increase in mean asymmetry, when considering the “subset” were the oVEMP and LARP tests. The metrics showing a negligible shift in mean asymmetry were instead cmpAP and SVV. Pearson’s test revealed a positive linear correlation between mean asymmetry values and the percentage of correspondence across all laboratory tests (R= 0.66 and p-value= 0.0194).

Regression-based predictions of the affected side. The vestibular laboratory tests were assessed for their relative predictive ability at identifying the affected side through different classifiers. To do this, Naïve Bayes, SVM and Logistic Regression were compared. As Logistic Regression showed a relative advantage over the other methods (see Supplementary Table 3), only this method’s results are shown for simplicity. The highest AUCs (all >0.9) were reached, in order, by the caloric, cVEMP and RLLL tests (Fig. 4).

Next, we explored the classificatory ability of each individual metric in a stepwise fashion, through a multiple regression analysis. To this end, an SVM was employed to assess the weights of the different metrics and rank them accordingly. The plot (see Fig. 5) indicates that after considering the caloric, cVEMP and RLLL tests, there was no predictive advantage in considering additional metrics (i.e., no further AUC increase).

Fig. 6 shows that the predictive ability of the SCCs’ asymmetry metrics taken together (RLLL, RALP and LARP) is similar to the predictive ability of the otolith’s asymmetry metrics combined (cVEMP and oVEMP), showing comparable AUCs.

## IV. Discussion

When a vestibular dysfunction is suspected, a battery of vestibular laboratory tests (including vHIT, VEMP, SVV and caloric tests) is typically conducted to quantify the peripheral vestibular asymmetry. The relative reliability and informative ability of such vestibular laboratory tests in helping a physician to identify the side of unilateral vestibular hypofunction can sometimes be challenging in clinical practice. In this study, we considered applying MLAs to estimate the predictive accuracy of such laboratory tests at identifying the VS side, whose true affected side was known (MRI-given). Specifically, in this study, we accomplished three main analytical steps: (1) Data pre-processing to clean up and prepare our data for analysis. (2) A statistical descriptive analysis to compare the laboratory tests’ mean asymmetry metrics in terms of size (i.e., how large an asymmetry is) and +/− direction (i.e., left or right asymmetry). (3) Logistic and multiple regression models were then applied to evaluate: (A) the relative predictive ability of each individual asymmetry metric (i.e., value and direction) alone and (B) the predictive ability of such metrics in different combinations, in a stepwise fashion. The model predictions (i.e., class labels) were finally compared to the MRI-given true labels to estimate the model’s accuracy and evaluate the relative utility of all laboratory vestibular tests.

Our results highlight the analytical challenges associated with the use of monocular vHIT, which have important clinical implications. The monocular vHIT introduces a side asymmetry in the VOR gain measurements that favors the measured eye [40], [41]. This left-right bias in the lateral canals’ VOR gains was attributed to the differences in the length of neural pathways between the ipsilateral and contralateral rectus nuclei [41]. Consistently, our data showed that the right vHIT gains (i.e., of the measured eye) were generally higher than the left ones, for both VS and comparison groups. This measurement bias would cause over- or underestimation of any identified asymmetries in the right and left VOR gains of VS patients, hindering accurate clinical conclusions. Furthermore, we observed that this bias concerns not only the lateral canals, but also the anterior and posterior canals [40], [41]. Therefore, to obtain a realistic and stable estimate of the asymmetry between the left and right VOR gains in VS patients, it was therefore necessary to develop a way to eliminate this inherent bias retrospectively. We proposed a computational approach that considers a normally performing group’s mean left-right difference in gains to eliminate intrinsic bias. While conceptually correct, our approach was limited by the fact that we had no readily available vHIT data from an age-matched healthy control group to perform such correction. Instead, vHIT data from a normally performing VM group (based on clinical observation) were used as a reference for approximating the size of the bias and eliminating it from the VS patient group’s data. A follow-up work should ideally consider using data from a large and age-matched healthy control group to ensure an accurate estimation of the gain bias introduced by the monocular vHIT test.

After correction, our results exhibited a visible change in the distribution of right and left VOR gains (relative to before correction) in schwannoma patients. For the lateral and anterior canals, a significant rightward shift is evident. In contrast, for the posterior canals, the difference was not as pronounced as for the other canals, although we observed a slight change in the tails of the distributions. There are three possible explanations for the difference between the vertical and horizontal data. First, the anterior canals are less orthogonal relative to each other and closer to the sagittal plane compared to the posterior canals. Hence, the vertical vHIT faces more challenges at separating the gains [48], [49]. Second, passive movements of the neck in the horizontal plane involve lower resistance compared to the movements in the vertical plane, easing the performance [50], [51], [52]. The last explanation might reside in a lower mean acceleration difference, which is a consequence of the second point discussed. Strikingly, after bias correction, most of the original statistical differences between the left and right gains of the VS patient group were no longer significant. This suggests that a large extent of left-right asymmetry in the gains was indeed attributable to the bias introduced by monocular vHIT, and not to the pathology itself. These findings have extremely important clinical implications, considering that diagnoses, prognoses and interventional decisions are partially based on vHIT results. This observation raises the necessity of applying data management and control measures, which we will discuss:

(1) The diagnosis of semicircular canal hypofunction should never be based on vHIT gain values alone, but always on the combination of a reduced gain plus the presence of corrective saccades [4], [53], [54], [55], [56], [57].
(2) One might furthermore consider introducing a computational tool that automatically adjusts the monocular vHIT gains, eliminating the bias. In this work, we have proposed a retrospective method to do so. However, as we discussed earlier, our approach was limited in that data of VM patients was used to correct for the bias. The diagnosis of vestibular migraine requires, among other criteria, the absence of pathological findings on auxiliary vestibular testing. Instead, data of a large and age-matched group of healthy control individuals would be necessary to obtain a reliable estimation of the bias (i.e., reliable “normative” values of the gain bias). However, the burden associated with the extensive battery of vestibular examinations was considered unacceptable for individuals without a direct clinical indication for undergoing these tests.
(3) Moving the fixation target on the wall further away from the subject might also help reduce the bias, as this would minimize the difference in fixation angles.
(4) Using a binocular vHIT may offer greater precision and accuracy than the monocular vHIT [58], and would be a preferable approach, as it would bypass the need to apply retrospective corrections.

On a final note, the LARP and RALP metrics are worth considering with regard to an additional aspect. While they are analogous to the lateral canals in that an excitation on one side produces an inhibition on the contralateral side, the RLLL measure selectively assesses the lateral canal’s functionality, while the RALP and LARP measures combine two different structures (ipsilateral anterior canal and contralateral posterior canal), which are functionally distinguished. Hence, LARP and RALP could be considered as being more mixed metrics than the RLLL. While it is possible to obtain a selective quantification of the lateral canals’ functionality by means of caloric testing, there is currently no technique providing an isolated measure for the anterior and posterior canals (i.e., LARA and LPRP). The introduction of the measure (cmpAP) was primarily aimed at obtaining a “composite measure”, where the left anterior and posterior canals are compared to the right anterior and posterior canals, which could be conceptually more relatable to all other asymmetry metrics (i.e., VEMPs, SVV, RLLL etc.).

Our descriptive statistical analysis shows the mean asymmetry values of the different laboratory vestibular tests and the side correspondence (i.e., in terms of asymmetry direction or sign) with the MRI-given true labels. While being only descriptive, the plot in Fig. 3 hints at the plausible predictive capacity a laboratory test might have. Specifically, when an asymmetry metric is large and matches the asymmetry direction of the pathology side (MRI-given), this will be expected to achieve a high sensitivity. The three metrics that fall in this category, based on Fig. 3, are the caloric, cVEMP and oVEMP tests. To extend our descriptive analysis, we further selected a subgroup of subjects for which the asymmetry direction identified by the laboratory tests matched the MRI-given tumor side (i.e., the so-called “subset”), computing the mean asymmetry value for all vestibular laboratory tests. This means, in the subset, we excluded those subjects for which the asymmetry direction did not correspond to the MRI-given affected side. The results highlight that the asymmetry values in the subset are higher compared to those in the whole VS patient group dataset. Metrics for which the increase in asymmetry values is most pronounced are the oVEMP and LARP. This might indicate the presence of “noise” in the whole dataset, meaning that the asymmetries identified by the laboratory vestibular tests are, in some cases, not explainable by the VS pathology. For the same reason, more consistent metrics (i.e., those that do not show a substantial shift in mean asymmetry) are, arguably, more reliable (e.g., SVV, cmpAP). Best metrics overall are those that show a high mean asymmetry and high consistency at the same time (i.e., caloric).

Our regression analysis next explored the relative ability of our different asymmetry metrics (i.e., laboratory vestibular tests) at predicting the affected side, using the MRI-given true labels. A logistic regression was first conducted to evaluate this at the individual metric level, while a multiple regression showed the predictive performance of different combinations of the tests. Paired bootstrap comparisons demonstrated that, in the univariate setting, several individual tests (RALP, LARP, SVV, cmpAP, and oVEMP) exhibited significantly lower AUCs compared with the caloric test. In contrast, RLLL and cVEMP did not differ significantly from caloric. On a general level, the results indicated that, in order, caloric, cVEMP, RLLL and oVEMP had the best predictive ability (Supplementary Table 3). This validates our preliminary descriptive statistics plot (Fig. 3), which revealed that these four metrics had the highest combined percentage of side correspondence and mean asymmetry values. Interestingly, the caloric test, which is a very low frequency test, was the best predictor and hence better at identifying VS than the vHIT [5], [59]. There are two possible explanations for this: First, the VS might first affect the vestibular nerve fibers transmitting low frequency information as tested by caloric irrigation (~0.003 Hz), while the fibers sensing high-frequency rotatory acceleration (up to 5 Hz) and assessed by the vHIT might be affected at later stages of the disease [23]. Second, the presence of secondary endolymphatic hydrops [60] in VS might cause a dissociation of intact vHIT and reduced caloric responses, as is commonly observed in Menière’s disease (i.e. primary endolymphatic hydrops [18]).

Unsurprisingly, the caloric and RLLL metrics, which both assess the lateral canals’ functioning, proved to be effective predictors of tumor side, with the first showing a relatively higher capability. Some functional differences, which might underlie a different predictive ability, can be considered between the two tests: (1) In the vHIT maneuvers, a given canal and the contralateral counterpart (LL with RL, RA with LP, LA with RP) are simultaneously tested. In contrast, the caloric test assesses one lateral canal at a time, excluding any side interactions or compensations. (2) As a high-frequency test, the vHIT is conventionally preferred, in that with some CNS conditions, such as vestibular neuritis (VN), it has superior clinical sensitivity due to its ability to capture pathologies in both chronic and acute stages.

Interestingly, the results show a similar intermediate predictive performance for the RALP and LARP metrics, whereas the composite measure cmpAP (as indicated by our descriptive plots) has the lowest AUC. This implies that the composite measure of the anterior and posterior semicircular canals lost relevant information. These predictive limitations are consistent with the observations of our descriptive plots. The SVV ranked second lowest in terms of predictive ability in both our logistic and multiple regression analyses, aligning with our descriptive plots, which showed a relatively low percentage of side correspondence and mean asymmetry value. These outcomes might be explained by the liability of the SVV to central compensation mechanisms, which can restore performance to control-like levels over time, reducing its discriminative ability [61].

The combined cervical and ocular VEMP metrics achieved similar predictive ability as the vHIT metrics when taken as a whole (Fig. 6). However, when assessed individually, VEMP metrics were superior to LARP and RALP metrics. This supports the notion that VEMP metrics are clinically important and should not be considered, on a diagnostic level, secondary to the vHIT metrics. The VEMP metrics remain central for understanding the effects of the tumor on the otolith’s functionality [62].

## V. Conclusion

MLAs offer valuable assistance to clinicians in interpretation of the results of multiple laboratory tests. In our study, three machine learning classifiers were compared for their performance. The regression-based classifier showed a relative advantage and was hence chosen to estimate the predictive capacity of different laboratory vestibular tests at identifying the side of VS, which was known (MRI-based). We have shown that, in order, caloric, cVEMP, oVEMP and RLLL tests achieved the highest predictive capacity and consistently exhibit a high mean asymmetry value and a high degree of correspondence with the MRI-given affected side. The better predictive ability of caloric, relative to the vHIT, in identifying the tumor side might suggest that, in VS, the slowly acting fibers in the vestibular nerve are mostly affected. Overall, the most robust predictive capacity was obtained by combining tests of the otoliths and semicircular canal functioning. Here, caloric and cVEMP tests alone achieved a combined AUC of 0.96. The caloric and VEMPs metrics have an important predictive capacity and critical functional screening role and should hence not be considered secondary to vHIT metrics in the context of VS. Caloric tests might be particularly helpful when the superior vestibular nerve is affected in VS [63], although this might not be generalizable to other vestibular pathologies.

Complementing MRI for the structural identification of the tumor with VEMPs, vHIT and caloric laboratory tests for screening the semicircular canals and otoliths’ functionality remains the most effective approach for an accurate clinical profile and treatment planning. Some further take-home points that we wish to highlight are as follows:

1) The gain bias in the lateral canals associated with the use of monocular vHIT goggles has been described before [40], [41]. In our study, we observed that the bias also applied to the posterior and anterior canals, although to a minor extent. Our findings support the need to account for this bias in clinical settings to ensure an accurate estimation of SCC functioning. We have discussed different control measures that could be undertaken. In this study, we have applied a computational method to retrospectively eliminate such bias. However, a follow-up study–using a larger and age-matched healthy control group as a reference–would be required to further validate our findings.
2) Mixed measures (i.e., RALP and LARP, combining anterior and posterior canals) seem to be disadvantaged at the predictive level compared to the non-mixed measures, especially caloric, RLLL and VEMPs). The composite measure of RALP and LARP (e.g., cmpAP), while conceptually promising, has shown no predictive advantage over RALP and LARP.VI.

## Author Contribution

Data analysis (Giorgia Rita Di Ruggiero and Carolina Beppi); drafting and writing of the manuscript (Giorgia Rita Di Ruggiero and Carolina Beppi), results interpretation (Giorgia Rita Di Ruggiero, Carolina Beppi, Christopher J. Bockisch, Sarah Hösli, Dominik Straumann, Julia Dlugaiczyk), revision of the manuscript for intellectual content (Giorgia Rita Di Ruggiero, Carolina Beppi, Christopher J. Bockisch, Sarah Hösli, Julia Dlugaiczyk, Dominik Straumann); data collection (Carolina Beppi, Sarah Hösli, Julia Dlugaiczyk, Dominik Straumann); study supervision (Carolina Beppi, Sarah Hösli, Dominik Straumann).
